# Supplementary figures and images for: The genomic and epidemiological virulence patterns of Salmonella enterica serovars in the United States
Source: PLoS One. 2023 Dec 5;18(12):e0294624. doi: 10.1371/journal.pone.0294624 (PMC10697515; doi:10.1371/journal.pone.0294624)

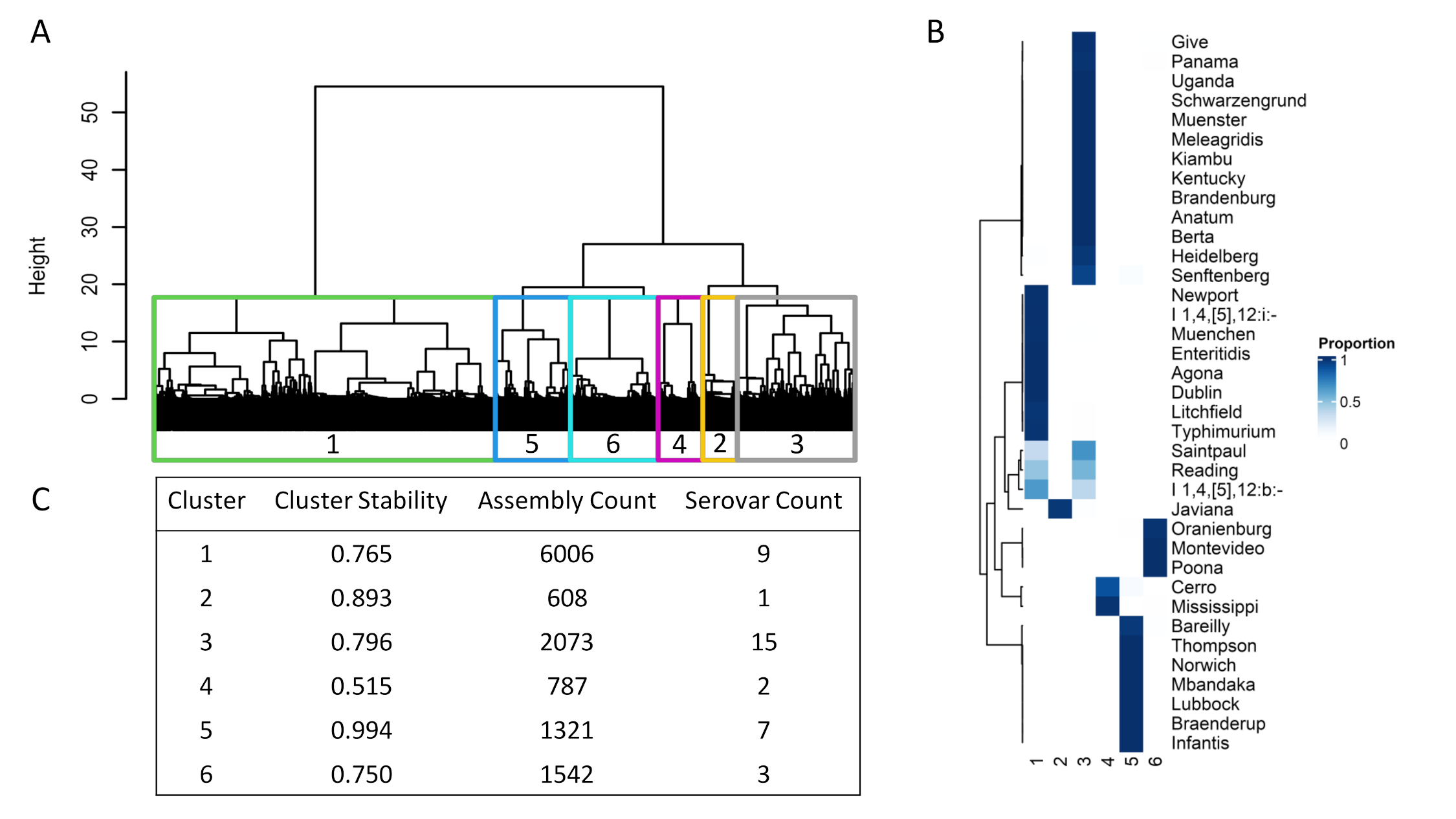

Supplement: S1 Fig — (A) Dendrogram depicting the hierarchical relationship between 12,337 S. enterica genome assemblies based upon virulence factor gene carriage with six virulence clusters superimposed on top. (B) Heatmap of serovar proportion within each of the six respective virulence clusters. Rows are clustered using Ward’s method. (C) Characteristics of the six virulence clusters: cluster stability—Jaccard similarity of 10,000 non-parametric bootstraps, Number of Genomes—depicting the number of S. enterica genomes constituent in each cluster, and number of serovars (within cluster serovar proportion > 0.5) in each cluster. (TIF) [file pone.0294624.s001.tif]
